# A Wireless Sensor Platform for Beehive Monitoring

**DOI:** 10.3390/s26061846

**Published:** 2026-03-15

**Authors:** Sudipta Das Gupta, Jeffrey Erickson, Joseph Rinehart, Benjamin D. Braaten, Sulaymon Eshkabilov

**Affiliations:** 1 Department of Agricultural and Biosystems Engineering, North Dakota State University, Fargo, ND 58105, USA; sudipta.gupta@ndsu.edu; 2 Department of Electrical and Computer Engineering, North Dakota State University, Fargo, ND 58105, USA; jeffrey.erickson@ndsu.edu (J.E.); benjamin.braaten@ndsu.edu (B.D.B.); 3 USDA—Agricultural Research Service, Fargo, ND 58102, USA; joseph.rinehart@usda.gov

**Keywords:** honey bees, beehive, temperature, humidity, CO_2_, honey bee stress, printed circuit board, remote monitoring

## Abstract

Honey bees are very important to the ecological environment and human society, contributing significantly to biodiversity and global food security, with an estimated annual impact of $15 billion in crop pollination in the USA. Over 62% of honey bee colony decline has been observed between June 2024 and February 2025. This study investigates bee stress level monitoring due to external disturbances like mechanical vibrations by measuring internal air temperature, relative humidity, and CO_2_ gas concentration levels of beehives. A new wireless sensor board for real-time monitoring of honey bee colonies was designed, built, and validated. The board incorporates NDIR-based SCD30 and SCD41 sensors for CO_2_, temperature, and humidity monitoring, integrated with a custom-designed two-layer printed circuit board and a Particle Argon^TM^ microprocessor for Wi-Fi communication. The developed board was tested and validated with live beehives in summer and winter of 2024 and 2025. The experimental study results showed the adequacy of the built sensor board. Bee colony responses on the applied stimuli (knocks) show that bees responded with a temperature increase of over 5 °C, CO_2_ concentration increase by 3000 to over 10,000 ppm, and, at the same time, relative humidity drop by about 10% inside beehives.

## 1. Introduction

A national study of beekeepers revealed significant losses of honey bee colonies throughout the United States, with commercial operations experiencing an average decline of 62% in their colonies from June 2024 to February 2025 [1]. These losses are significantly greater than average year-to-year declines and might have serious consequences for US agriculture, particularly for products that rely heavily on pollination, such as almonds, fruits, and vegetables.

Honey bees play a vital part in world agriculture by pollinating various crops. Honey bees contribute substantially to both ecological stability and food production systems through their role as key insect pollinators [2,3,4]. Bee pollination services in the U.S. are estimated to be worth $15 billion per year [5].

However, pollinator diversity is reported to be declining. Reductions in pollinator variety and abundance can affect the reproduction of plant species, crop production, food security, and human well-being [6]. With increasing reliance on managed honey bee colonies for pollination services, especially in large-scale agricultural systems, there is a growing need to ensure the health and well-being of these vital pollinators. However, honey bee colonies are subjected to a variety of stresses, particularly during winter storage and movement, which can impair colony health and diminish pollination performance. Monitoring honey bee stresses in real-time using technology is a proactive way to address difficulties and promote sustainable agricultural productivity [7,8,9,10].

In many agricultural contexts, including apiculture [11,12,13,14], wireless sensor networks (WSNs) are increasingly used for real-time environmental monitoring [15]. These systems support continuous remote data collection and offer critical insight into hive conditions, such as temperature, humidity, and CO_2_ levels [16]. When combined with Internet of Things (IoT) technologies, WSNs also improve monitoring, prediction, and diagnostic capabilities [17]. In this study, honey bee colonies were monitored using a cost-effective and scalable WSN.

Some studies addressed the deployment of WSNs in contrast to other wireless solutions and commercial systems; however, those are too expensive for pollination managers to employ in large quantities [18]. Despite their potential, existing WSN technologies are usually costly and energy-intensive, which makes adoption challenging in resource-constrained beekeeping applications. Among these resources, the main WSN challenge is power management [19]. Energy consumption has a direct effect on operational longevity of sensor nodes, especially in remote or resource-constrained settings where frequent battery replacement or recharging is not feasible. Duty cycling and energy harvesting are only two of the energy management techniques that IoT networks have embraced [20]. Due to changing environmental circumstances and the particular design limitations of hive-based monitoring systems, these techniques might not always be appropriate for particular apicultural environments. Therefore, by providing continuous monitoring of crucial stress factors like hive temperature changes, relative humidity, and CO_2_ accumulation, low-cost, energy-efficient WSNs designed for apiculture could completely transform the industry. This innovation could support colony health and productivity by enabling beekeepers and researchers to monitor important stress factors and perform regular maintenance. Additionally, they might provide extensive research on how pesticides, climate change, and other environmental stresses affect the behavior and health of honey bees.

Whether in the city or the remote countryside, beekeepers are becoming more interested in using sensors in their hives, which can be explained by the advancement of IoT technology [21,22,23]. Among other hive variables, temperature, humidity, and concentration of CO_2_ are important ones [24]. It is also possible to quantify environmental characteristics based on hive location, such as temperature, humidity, wind, and rainfall [25]. Research in this direction is growing along with the studies demonstrating various approaches to monitoring honey bee stress factors. For instance, some studies have focused on high-sensitivity sensors that capture detailed environmental metrics, while others emphasize the practicality of more energy-efficient and affordable sensor designs suitable for field deployment. A relatively cheap solution was developed, simple in operation and a robust solution for remote swarming detection, and one temperature sensor per colony was installed above the upper hive body to get sufficient information to recognize swarming events [26]. There is ongoing debate regarding the balance between sensor accuracy and energy efficiency; some studies are underlining comprehensive monitoring solutions, and others are supporting simpler, more scalable systems such as ultra-wide band radar sensors [27]. These differing perspectives highlight the complexity of designing WSNs that are both functional and feasible for wide-scale use in beekeeping [28]. Studies [29,30] include weight alongside temperature continuous monitoring of honey bee colonies.

An important issue is the power source in the apiary location; if there is no AC power connection available, then alternative power supplies should be considered (e.g., solar energy) [31]. In scenarios where solar power is employed alongside wireless technology for data transmission [32], it is essential to consider reducing the volume of data transferred from the measurement node to the remote server to conserve energy [33]. Researchers have explored various approaches to monitor beehives. Ferrari and others used computer sound cards with omnidirectional microphones while tracking temperature and humidity [34]. Chen et al. developed an imaging system at hive entrances, using tags to identify and count individual bees [35]. Zacepins et al. focused on winter monitoring by tracking hive temperatures and applying algorithms to determine colony status [36]. Heidingeret and others employed RFID technology to study queen bee mating flights [37]. Kviesis et al. developed a wireless system with a main unit that communicated with sensors in each hive and served as an internet gateway [38]. They monitored temperature and humidity using SHT15 sensors and transmitted the data to a remote server. Zacepins et al. created a temperature monitoring system using Raspberry Pi as an internet gateway, connecting hive sensors through a one-wire network—a cost-effective solution when using a single Raspberry Pi for multiple hives [39].

Murphy and others designed a WSN with two waspmote (open-source wireless sensor platform) nodes per hive, measuring various parameters including temperature, CO_2_, and pollutants at six samples per day. Data was stored on SD cards and transmitted daily via Zigbee protocol to a base station, which forwarded it through 3 G/GSM to a remote server. They later developed algorithms to detect colony threats and even predict weather conditions based on CO_2_ levels [10]. Kridi, Carvalho, and others [40] built an Arduino-based system using an LM35 temperature sensor and XBee-Pro wireless transmission to transfer sensor data to a base station. The collected temperature sensor data helped them to identify thermal stress patterns leading to colony abandonment. Jiang and others [9] developed a system tracking bee entrance/exit frequency along with internal and external temperature and humidity. This attempt was innovative, and bee counting accuracy varied within hive activity and resulted in an average of 10% error and maximum errors of 20%.

The promising advancements in beehive monitoring technology face challenges that limit their widespread implementation and long-term durability. These systems encounter particular barriers when deployed in remote bee farms with resource constraints, where considerations of reliability, cost, and maintenance become critical factors for practical application. Unfortunately, most of the solutions mentioned above are not low cost, not viable for mass application and not energy efficient, which is not good for remote applications [41]. Most of them were commercial high-cost devices, and the use of wired sensor systems, as opposed to wireless technologies, has been reported to interfere with the natural behavior and comfort of honey bees within the hive. Additionally, these are not energy-efficient, a critical drawback for applications requiring sustainability and independence from conventional power sources [15,16,42]. Bees are sensitive to any foreign objects [43,44] inside their hive. Any wired uncovered sensors and systems inside the beehives are intervened with by bees, which affects the quality of data and raises questions regarding the vulnerability of the system, the accuracy of data, and the reliability of the system [45].

The core objective of this study is to develop and implement a low-cost, reliable, and energy-efficient wireless sensor system using SCD30 vs. SCD41 sensors for monitoring honey bees inside beehives. The developed sensor system provides real-time monitoring of internal beehive variables, such as temperature, relative humidity, and CO_2_ level fluctuations to observe bee responses on external disturbances. The performed tests during forage and hibernation periods with live beehives showed the practical feasibility of the developed system and its monitoring accuracy to detect abnormalities inside beehives.

**Temperature.** Although bees are considered ectothermic animals [46,47], they exhibit temperature self-regulation characteristics when functioning as a group. Honey bee receptors are capable of sensing as low as 0.2 °C temperature change [48] and try to maintain certain temperature conditions inside their hives, particularly near the bee larvae areas [48]. Worker bees try to keep the brood temperature within 34.5 ± 1.5 °C to safeguard optimal larval development and colony function [49,50]. Thermoregulation of the bee colonies within 34.5 ± 1.5 °C range is critically important for the pupae and larvae and adult bees as well [51,52,53,54,55]. Therefore, bees keep regulating the brood nest and hive temperature within this range. Some studies show that staying a long time below 32 °C temperature results in a high risk of maldevelopment of body parts of larvae and neural and behavioral insufficiencies of adult bees [51,52,53,54,55]. For example, the study [54], carried out with long-distance transportation of beehives for pollination purposes, shows that the airflow and temperature below 32 °C caused significant stress on bees, or even smaller bee colonies never recovered from the caused stress, and also, many bees died. Honey bees are not only sensitive to temperature changes but also to the air quality in their hives. The studies [56,57] show that artificial interventions, such as heating or cooling, may help to reduce colony losses due to temperature imbalances inside a beehive.

**CO_2_ Concentration.** Honey bees sense and react to CO_2_ concentration changes in their hives to maintain a CO_2_ concentration below 11,000 ppm by fanning out the air from their hives [48]. According to the studies [58,59], normal in-hive CO_2_ concentrations range between 1000 and 5000 ppm and may go up to 10,000 ppm or even higher under conditions of poor ventilation or crowding. Elevated CO_2_ levels reflect conceded hive ventilation, clustering, or colony stress responses and may serve as an early indicator of disturbance or behavioral change in a bee colony [59,60,61,62].

**Relative Humidity.** The relative humidity inside the hive generally ranges between 40 and 70%, considerably influenced by ambient weather and colony activities [63,64,65]. The relative humidity is particularly critical for egg hatching and survival, and optimal relative humidity was observed to be 90 to 95% [66,67]. Abnormally high or low relative humidity levels impact brood development and create stress in the bee colony [66,68].

The experiments were conducted in three different hives at USDA–ARS, Fargo, North Dakota, to ensure controlled deployment and validation of the system by the researchers of North Dakota State University and USDA–ARS in collaboration. We tested the benchmarking PCB with SCD41 sensor at USDA–ARS facilities with three healthy honey bee (Apis mellifera) colonies (estimated 50,000–60,000 workers; worker age from 2 days to 2 months, mostly ~1.5–2 months; queen sourced from the same apiary and season). In these experiments, effects of regional variations in climate and nectar sources were not considered. The studies were aimed at validating the designed PCB’s performances under two contrasting environmental conditions—during summer forage (summer of 2024 and 2025) and winter hibernation (winter 2025).

The proposed system architecture allows a noble non-invasive placement decision (“Frame” vs “Bottom”) tied to colony activity and physical constraints and an in-hive paired comparison of the SCD30 vs SCD41 vs HOBO CO_2_ data logger, reporting Pearson r and bias and explaining offset propagation via T, RH and CO_2_. Our proposed system presents an advancement in non-invasive monitoring techniques for social insect colonies with energy vs sampling-rate design tradeoff, including multi-rate tests and a battery discharge model (R^2^ and RMSE), which is noble in this domain of study. The specific contributions of this work are as follows:

- A novel energy-efficient PCB-based beehive monitoring system with three sensing variables was developed.
- A developed PCB-based beehive monitoring system was tested and validated for accuracy and energy consumption in live beehives in different seasons of the year.
- A battery discharging process of the developed PCB was modeled.
- A benchmarking analysis of performance characteristics of two sensors, NDIR SCD30 and SCD41, in live beehives was performed.

The following sections of the paper are structured as follows: Section 2. Materials and Methods describes beehive assessment and proposed PCB design; Section 3. Wireless Sensor Network and Model Development elaborates the wireless data communication set up, sensor integration, experimental setup and voltage drop modeling issues; Section 4. Results and Discussion section displays experimental study results, sensor comparison analysis, and discussions of the obtained results; and Section 5. Conclusions summarizes essential findings and developed works and future work plan.

## 2. Materials and Methods

The PCB measurement system was developed with careful consideration of the physical and technical constraints and design criteria required to minimally disrupt the daily activities of the bees across all seasons—winter hibernation and spring and summer foraging.

### 2.1. Beehive Assessment

Prior to establishing the measurement setup, a team of expert entomologists from the USDA–ARS in Fargo, ND, assisted in assessing the various beehive models used in this study. This evaluation involved measuring the internal dimensions of the beehive, identifying key areas where bees predominantly congregate and engage in essential activities, and assessing the overall functionality of the hive. The purpose of this assessment was to identify optimal sensing locations and strategically position the hardware and sensor systems inside the beehive according to the design criteria. Based on this assessment, two primary points of interest were selected, focusing on areas where the bee colony actively engages, particularly during winter hibernation. These locations include (i) the space between frames, near the brood area (“Frame”), and (ii) the bottom section of the beehive, where bees move between frames and where any deceased bees are collected on the bottom plate (“Bottom”). Monitoring at the entrance was excluded due to the minimal bee activity observed during winter months. The generic wooden beehives were used in this study.

Knocking on the beehive serves as a non-invasive stimulus intended to elicit a measurable behavioral and physiological response from the colony. Studies [69] showed that bees not only communicate via their vibroacoustic sensory organs but also sense any abnormal external disturbances such as mechanical vibrations. Such mechanical disturbances are known to trigger alarm and defensive behaviors, including increased movement, wing fanning, and ventilation activity. These behaviors can result in short-term changes in internal parameters. CO_2_ levels may increase due to elevated respiration rates from agitated bees. There may be changes in temperature and humidity due to collective muscular activity and in airflow caused by increased wing fanning or clustering responses of bees. Observing how these parameters fluctuate post-disturbance and how quickly they return to baseline provides insights into the colony’s resilience, stress tolerance, and recovery dynamics. Colonies under chronic stress or poor health may show delayed or dampened recovery, which can be a valuable proxy for overall colony vitality.

Bee colony response variables, such as temperature, relative humidity, and CO_2_ concentration changes inside a beehive on external physical disturbances (knocks on the beehive frame from outside), were measured with the Adafruit’s NDIR SCD-30 and SCD41 sensors embedded into our latest developed printed circuit board (PCB) version 4 for a comparative analysis of power efficiency and reading accuracy of the two sensors. After installing the PCBs in beehives, we compared the accuracy of temperature, relative humidity, and CO_2_ from SCD30 and SCD41 (see Section 4). The technical specifications of the developed PCB are elaborated in the next section—Section 2.2.

### 2.2. Printed Circuit Board Development

**Figure 1:**
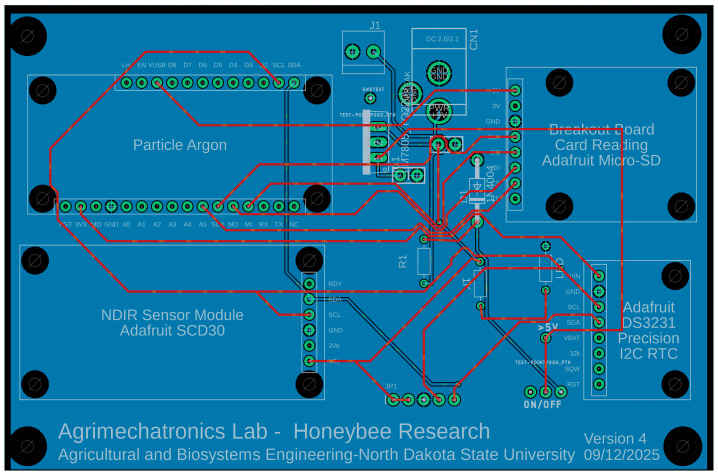
Designed PCB layout.

Considering the technical constraints outlined in Section 2.1, the experimental beehive was instrumented at two key locations—the brood area (“Frame”) and the bottom plate (“Bottom”). A multiparametric acquisition platform has been designed to monitor temperature, humidity, and CO_2_ gas concentration levels produced by honey bees within a hive. The printed circuit board (PCB)—shown in Figure 1—was designed using Autodesk Fusion 360, a commercial PCB design software application developed by Autodesk (San Francisco, CA, USA). The PCB incorporates a Particle Argon^TM^ (Particle Industries, San Francisco, CA, USA)—nRF52840 (Nordic Semiconductor ASA, Trondheim, Norway) with Mesh and Wi-Fi, developed by Particle Industries (San Francisco, CA, USA); an Adafruit SCD-30—NDIR CO_2_ (Adafruit Industries, New York, NY, USA)—Temperature, and Humidity Sensor, a MicroSD card breakout board, and an Adafruit DS3231 Precision RTC Breakout by Adafruit Industries (New York, NY, USA). For viable power supply options, the system can also be powered via USB through the Particle Argon^TM^ or with a 5 V adapter through a 2.0 mm DC jack. Additionally, other power supplies exceeding 5 V can also be used through a power jack, and for such cases, a switch is included to ensure component safety. The system was designed with low power consumption in mind, enabling a self-sustainable operation via a 9 V battery.

The PCB’s central component is a Particle Argon^TM^, which is a powerful Wi-Fi development kit. Most sensors were attached over the industry-standard SPI and I^2^C buses available on the Particle Argon^TM^. The system was designed in such a way as to minimize disturbance to the bee ecosystem and prevent the impact of direct exposure to the weather to avoid measurement errors.

Sensors inside the PCB were positioned in such a way that they would function without the danger of being obstructed by bee wax, as bees wax any foreign objects inside their hive to protect the colony from any intervention. The PCB’s collected data from the hive represent the microclimate of the living environment of bees and bee colony’s response characteristics for any considerable abiotic or abiotic stressors. The PCB components and their specifications are shown in Table 1.

## 3. Wireless Sensor Network and Model Development

**Figure 2:**
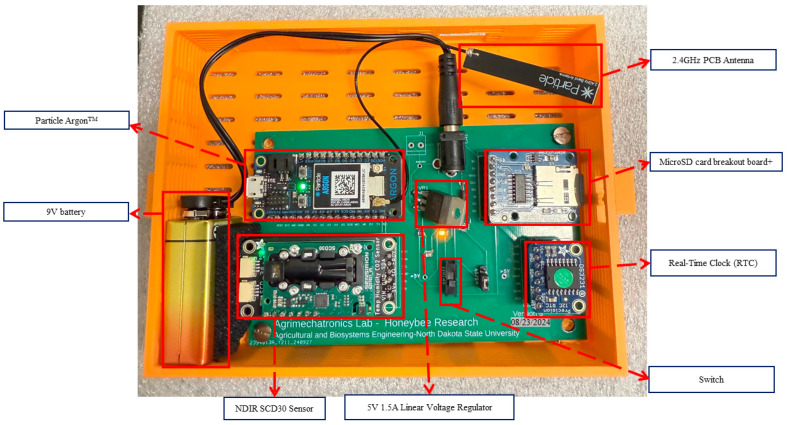
PCB (Version-3) built in 3D–printed custom housing and its main components.

The wireless sensor data acquisition and processing were designed to monitor the stress levels of honey bees in response to abiotic stressors, i.e., external disturbances. The system was based on the microcontroller-based platform and stored the data in a microSD, and the measurement data obtained by each node were transmitted to a local server and, from there, to the cloud database server via Wi-Fi protocol. In this way, we ensured that a beekeeper could monitor and download the sensor data of temperature, relative humidity, and CO_2_ inside a beehive and, more importantly, in a non-intrusive way without disturbing bees. Figure 2 shows the WSN product with 3D-printed housing. In the development of a sensor-based system for real-time environmental monitoring within beehive ecosystems, four distinct iterations of a PCB were performed. Each version reflects progressive refinements in functionality, precision, and practicality tailored to the specific requirements of apicultural research.

### 3.1. Benchmarking PCB

**Figure 3:**
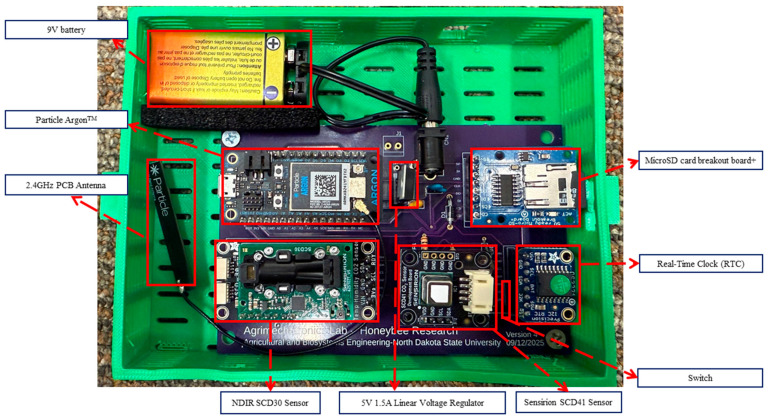
Benchmarking PCB built in 3D–printed custom housing and its main components.

The benchmarking version of the PCB (Figure 3) was designed with two Adafruit sensors (Adafruit Industries New York, NY, USA.)—SCD30 and SCD41, to compare the performances of these sensors. This benchmarking PCB was deployed in our field studies with live beehives.

**Figure 4:**
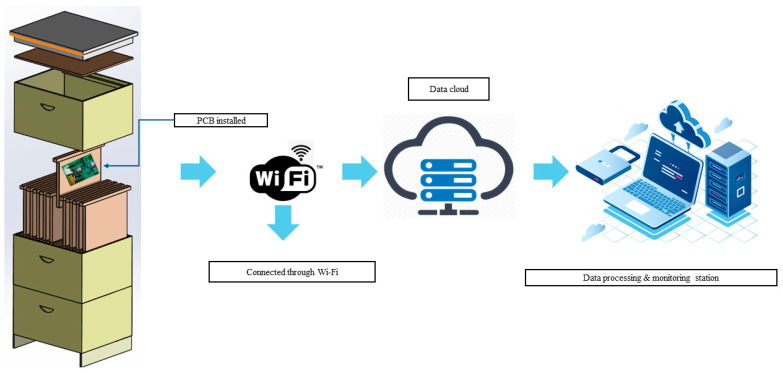
System architecture.

The entire system architecture is shown in Figure 4 that is composed of PCB, Wi-Fi communication, cloud storage and data processing. The system is controlled by the ARM Cortex-M4F 32-bit processor 64 MHz microprocessor (Arm Ltd., Cambridge, United Kingdom), which communicates with microcontrollers and initiates the collection of data that are forwarded via Wi-Fi to the cloud system and web database. The data is also written into local storage. The Particle Argon^TM^ platform includes the IDE (Integrated Development Environment) used to program particle devices. This IDE offers a series of function libraries to easily control the different peripherals, such as the microSD card, RTC, UART, and the digital I/O. The built PCB sets were installed in a few spots following the structure of frames and floor in the hive. To measure ambient conditions outside of the hive, one PCB was placed on the outer part of the beehive box.

### 3.2. CO_2_, Temperature and Humidity Sensor

An Adafruit NDIR SCD-30 sensor (Adafruit Industries, New York, NY, USA) was used to measure the CO_2_, temperature and humidity inside the beehives. This sensor was chosen due to its excellent reliability and stability and its low power consumption. Temperature is measured via a band-gap sensor module, relative humidity is measured through a capacitive sensor module, and both modules are integrated into this sensor—NDIR SCD-30. CO_2_ is measured using CMOSense^®^ Infra-Red technology (Sensirion AG, Stäfa, Switzerland). The sensor automatically compensates for long-term deviations due to the dual-channel CO_2_ concentration measurement principle. The sensor’s relatively small size (35 × 23 × 7 mm) minimized the overall dimensions of the designed PCB. According to the factory recommendation for continuous measurement with the SCD30 sensor (sampling interval from 2 s to 1800 s), the data acquisition speed is set to two seconds. This is the fastest limit for measuring and transmitting the sensor signals to the smart device. The sensor’s accuracy is ±30 ppm for CO_2_ concentrations from 400 to 40,000 ppm and ±50 ppm for CO_2_ concentrations above 10,000 ppm.

The Sensirion SCD41 photoacoustic NDIR CO_2_ sensor (Sensirion AG, Stäfa, Switzerland) was embedded on our latest PCB version (Figure 4) to monitor in-hive CO_2_, temperature, and relative humidity. The sensor supports continuous and single-shot measurement modes and offers a CO_2_ measurement range of 400–5000 ppm. Typical CO_2_ accuracy is ±50 ppm, with ±2.5–5.0% of measured value depending on conditions (best case ±40 ppm). The device operates from 2.4 to 5.5 V, with an average supply current of ~15 mA (max 205 mA during peaks) and an operating temperature range of −10 to 60 °C. In this study, we sampled the SCD41 readings at 5 s intervals to align with the rest of the acquisition stack.

### 3.3. Experimental Setup

**Figure 5:**
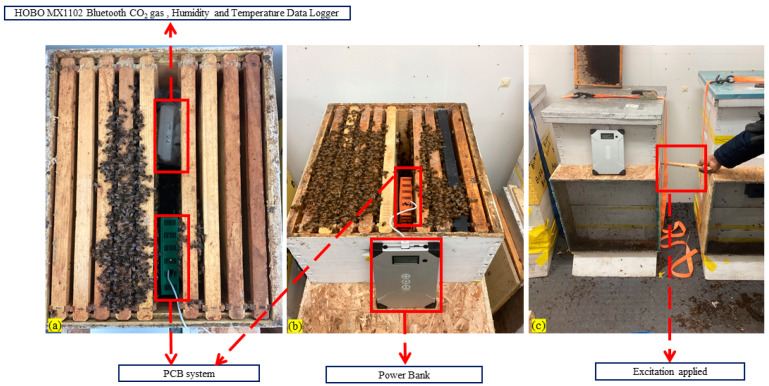
Experimental data collection setup of our designed PCB and HOBO data logger: (**a**) HOBO (top) and our PCB unit, (**b**) our PCB unit and power bank, (**c**) external induced stimulus (knock on the beehive) on 13 January 2025.

**Figure 6:**
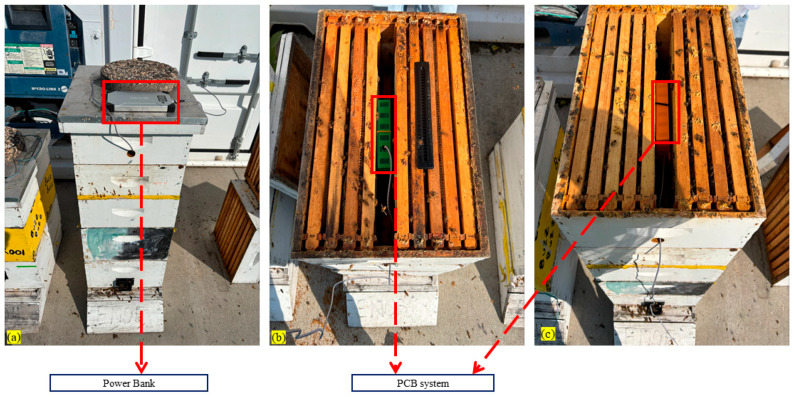
Experimental data collection setup of our designed PCB: (**a**) power bank, (**b**) our PCB unit in Hive-1, (**c**) our PCB unit in Hive-2.

Beehives typically accommodate 10 to 12 frames, which occupy the entire internal space of the hive. The space between bee combs inside a beehive is very tight, and an intra-frame spacing is roughly 17 mm. Inspection points, such as the top and bottom of the hive, also have limited space. Consequently, the design of the monitoring device and its allocated space must be sufficiently small to fit within the confined dimensions of the beehive. The prototype was installed in the middle of the beehive, where most of the bees concentrated. An active beehive with the PCB main unit and sensors used for data collection in this research is shown in Figure 5. For installing the PCB system equipped with a 3D box inside the beehive, we took out one empty frame from each side of the beehive and made enough space to accommodate the system in the middle of the beehive, as shown in Figure 5 and Figure 6.

Three active beehives with the PCB main unit embedded with an Adafruit NDIR SCD-30 sensor (Adafruit Industries, New York, NY, USA) and a Sensirion SCD41 photoacoustic NDIR CO_2_ sensor (Sensirion Inc., Chicago, IL, USA) used for data collection in this research are shown in Figure 6. The PCB was put inside a 3D-printed housing before installing it inside the beehive. The housing of the PCB was put with sufficient space to accommodate the system in the middle of the beehive, as shown in Figure 6.

### 3.4. Voltage Drop Analysis

The battery’s discharge (voltage drop) estimation V(t) over the operational period of the PCB at different data sampling rates was modeled as shown in Equation (1) and based on the total decay time ($t_{d}$) as a function of time [70]. The battery discharge process has three regions: region 1—a short nonlinear voltage drop at the start; region 2—long linear mid-discharge; and region 3—exponential decay at the end before complete drain of the battery’s charge.

(1) $$V\left(t\right)=V_{0}-V_{1}t-e^{-\left(V_{d}\left(t-t_{d}\right)\right)}+\varepsilon$$

where $V_{0}$ is the initial (nominal) voltage of the battery pack at the start of the test in region 1, $V_{1}\;$ is the voltage drop slope in region 2, $V_{d}$ is the voltage decay rate in region 3, and ε is the hardware related noise in the system. The values of $V_{0}\;and\;V_{1}$ are influenced by the discharge process cycle and internal resistance of the circuit components. Model accuracy was evaluated by comparing $V\left(t\right)\;$ against the measured voltage drop values using RMSE and $R^{2}\;$ in the linear discharge region as shown in Equations (2) and (3) [71]:

(2) $$RMSE=\;\sqrt{\sum_{i=1}^{N}\frac{{\left(V_{measured}-V\left(t\right)\right)}^{2}}{N}}$$

(3) $$R^{2}=1-\frac{{\left(V_{measured}-V\left(t\right)\right)}^{2}}{{\left(V_{measured}-{\bar{V}}_{Measured}\right)}^{2}}$$

where $V_{measured}$ is the measured voltage drop values during the actual test of the PCB and ${\bar{V}}_{Measured}$ is the mean value of the measured voltage drop.

## 4. Results and Discussion

### 4.1. Sensor Validation and Calibration

**Figure 7:**
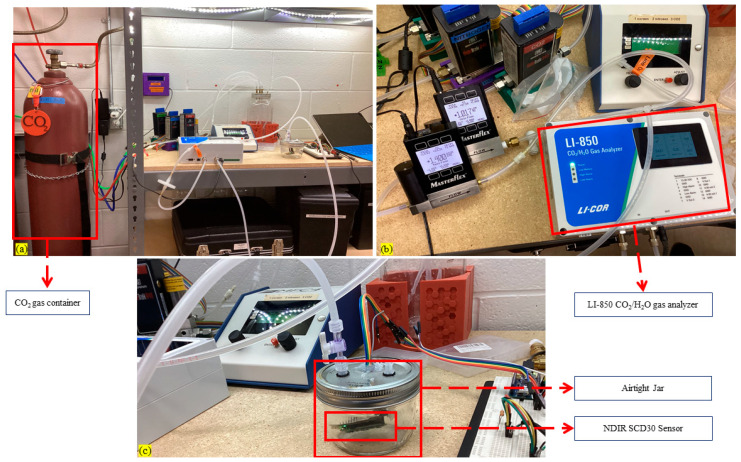
Calibration setup: (**a**) overall calibration setup, including CO_2_ gas container; (**b**) LI-850 CO_2_/H_2_O Analyzer device; (**c**) validation was performed by injecting distinct amounts of CO_2_ gas into the air-tight, sealed, cylindrical jars.

To identify the sensor’s suitability for in situ, dynamic closed-chamber measurements, the precision and accuracy of five sets of Adafruit NDIR SCD-30 sensors were tested and validated via laboratory experiments before implementing in the beehive experiment. Sensors were placed separately into a sealed, ventilated, cylindrical jar and connected to the developed data logger system, as shown in Figure 7a–c. All sensors were calibrated in ambient air before use according to the manufacturer’s instructions. Afterwards, different distinct amounts of CO_2_ gas were injected into the sealed jar—Figure 7a–c. In between injections, the jar was flushed with ambient air by two pumps connected to the jar. We injected five different CO_2_ gas concentrations into the jar and collected readings from an LI–850 CO_2_/H_2_O gas analyzer (LI-COR, Lincoln, NE, USA), forced recalibration (FRC) value, and our employed NDIR SCD-30 sensor readings and put all data in Table 2. CO_2_ concentration was increased inside the jar, measured with a 5 s interval by the NDIR SCD-30 before and after injection (CO_2_ concentration in ppm), and compared against induced CO_2_ concentration. All five measurement points from the LI–850 CO_2_/H_2_O gas analyzer and SCD30 showed a positive linear relationship with small offsets, i.e., 9–24 ppm or approximately 0.2 to 2% deviations from the true CO_2_ gas concentration values—Table 2.

The validation of temperature and relative humidity readings of the employed sensor—Adafruit NDIR SCD-30—was done by comparing the readings with a high-precision Onset HOBO MX1102 Bluetooth CO_2_, Humidity and Temperature Data Logger (Onset Computer Corporation, Bourne, MA, USA). The temperature readings of NDIR SCD-30 differed from the ones of HOBO by a consistent ~1 °C, and the relative humidity differed by ±2–3%.

### 4.2. Beehive Measurements

To evaluate the performance and reliability of the proposed beekeeping WSN system, tests were conducted in USDA–Agricultural Research Service, Fargo, ND, in September 2024, with summer bees during forage, and January 2025, with winter bees during hibernation. The details of the performed experiments with three different hives are shown in Table 3. There were 10 operator-induced knocks with a time interval of 5 s on the beehives to excite/disturb bees at night during the forage season when bees are less active, and on the contrary, during the hibernation season, the excitation knocks were performed during the day time. The sound power of the knocks was around ~65–75 dB. The excitation acceleration magnitude of the knocks on the beehive wooden house was not measured.

**Figure 8:**
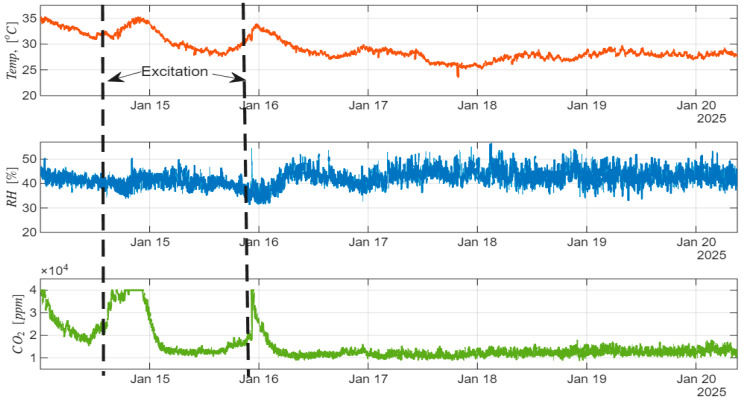
Sample data of temperature, humidity, and CO_2_ collected in January 2025. Dashed lines denote applied excitations.

Our winter experiments that were run in January 2025 in healthy beehives during the bee hibernation period showed that, by applying a disturbance on the beehive wall, the temperature increased by about 5 °C, and the CO_2_ concentration level increased up to 40,000 ppm (Figure 8), but the relative humidity change was not significant. This clearly shows the behavioral change in bees in response to the applied stimuli (knocks). Our observations with the beehive temperature change were found to be similar to the study results of [72,73,74,75]. Other similar studies, e.g., Meikle et al. [29,30], strategically positioned sensors in the upper region of the hive, while Giammarini et al. [76] opted for sensor installation on the internal surface of the hive’s foundational structure, deliberately avoiding direct placement on the frames. Critically, these alternative positioning strategies potentially compromise the fidelity and precision of empirical data collection as compared to the methodologically optimal on-frame sensor deployment strategy in [77,78].

**Figure 9:**
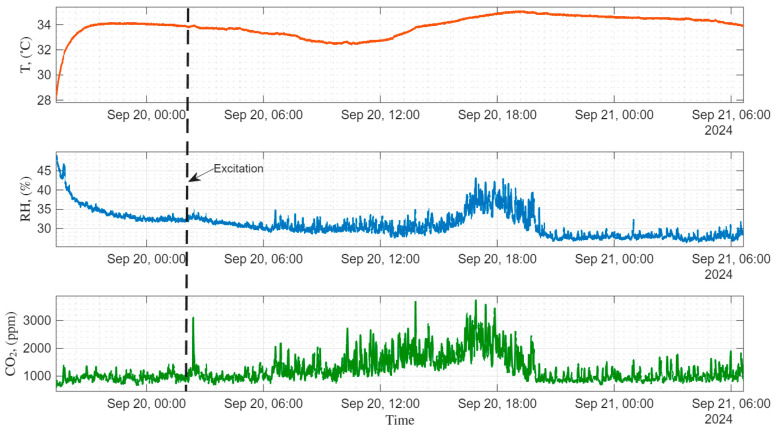
Test data of temperature, humidity, and CO_2_ collected in September 2024. Dashed lines denote applied excitations.

**Figure 10:**
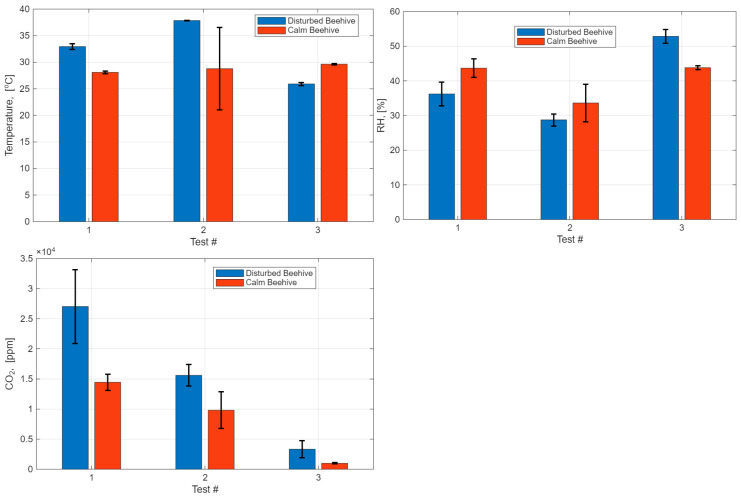
Statistical analysis of temperature, relative humidity (RH), and CO_2_ concentration changes inside the beehives after excitation (disturbed beehive) and calm beehives.

During the forage period in September 2024, we collected data on two successive days in healthy beehives. Measurements were performed with a 5 s sample time for 24 h. The experiments during the forage period (Figure 9) showed that, after applying a disturbance—a knock on the outer wall of the beehives—the temperature change inside the hive fluctuated by ~0.5 °C, which is not significant. Similarly, the relative humidity (RH) change was not significant. On the other hand, CO_2_ concentration ramped up from 1000 ppm to 3000 ppm, which indicates the change in microclimate inside the beehive—Figure 10.

The statistical analysis of the other three different experiments in three different healthy beehives shown in Figure 10 demonstrates the statistical significance of the bee responses on external excitation—stimuli. The temperature and RH changes inside the beehive due to the applied stimuli in the first two tests showed a direct correlation between excitation and bees’ responses (Figure 10). The correlation between the CO_2_ concentration level changes and applied excitation was positive in all three tests.

**Figure 11:**
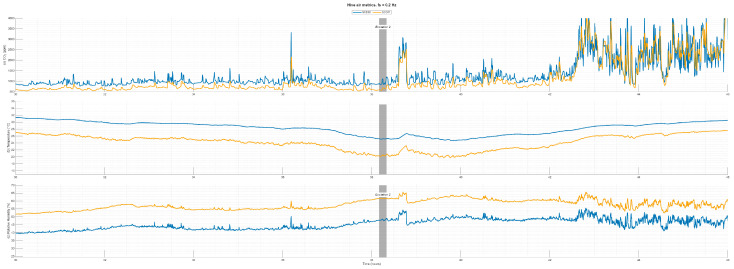
External disturbance effects on (**a**) CO_2_ gas concentration and (**b**) temperature and (**c**) relative humidity in both sensors’ data inside a beehive on 24 and 25 September 2025. Vertical grey bar denotes applied excitation.

For comparison purposes of the two employed sensors, SCD41 and SCD30, embedded in Ver 4 of our developed PCB, it was tested inside the live beehives for about 46 h. The readings of the two sensors were very close but with some consistent offsets—Figure 11. Across the full three-day record for both hives, co-variation was high: r ≈ 0.98 for CO_2_, 0.93 for temperature, and 0.95 for RH, indicating that the sensors tracked diel and excitation-evoked dynamics closely. Nevertheless, we observed sensor reading differences of SCD 30 and SCD41 of CO_2_ + 178.8 ppm (~3% of 6000 ppm scale), temperature offset −3.75 °C, and RH offset +11.87%, based on the test data shown in Figure 11. Given the SCD sensor’s internal T/RH-coupled CO_2_ compensation, offsets of this magnitude, especially around 3.8 °C and +11.9% of RH, are expected to propagate into CO_2_ estimates.

**Figure 12:**
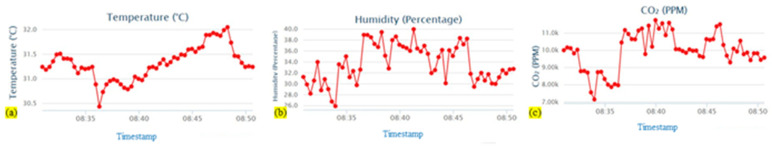
Cloud integration (ThingSpeak^TM^) to show the beehive condition dynamics: (**a**) temperature, (**b**) relative humidity, and (**c**) CO_2_ measurements.

Cloud-based data storage and visualization options were integrated into the proposed WSN system. All measured data, such as the hive’s internal temperature, relative humidity, and CO_2_ concentration levels along with corresponding timestamps, are systematically stored in a cloud database. These collected data could be shown in graphical format and tabular formats, with additional functionality of exporting data in .CSV files for offline analysis. Real-time data visualization is supported through dynamic graphs displayed directly within the web interface, offering immediate insights into hive conditions. The cloud infrastructure is implemented via ThingSpeak^TM^, (MATLAB R2024b, MathWorks, Natick, MA, USA) with a subscription, which facilitates data display through integration with MATLAB (MATLAB R2024b, MathWorks, Natick, MA, USA), as shown in Figure 12.

The presented discussion of temperature, relative humidity, and CO_2_ measurements is primarily framed within an environmental monitoring perspective; variations in these parameters are fundamentally coupled to changes in the dielectric permittivity and loss characteristics of the surrounding medium. Although the sensing approach employed in this study relies on direct measurement of environmental variables rather than electromagnetic interrogation, fluctuations in moisture content, gas concentration, and biological activity inherently modify the effective dielectric properties of air, wax, and surrounding biological materials within the hive. Moreover, recent studies have demonstrated that humidity, moisture dynamics, and complex biological compositions can be sensitively detected using planar microwave sensors and RF meta sensors based on dielectric dispersion and loss behavior [79,80].

Another important phenomenon in bee responses is their vibratory response patterns while communicating with each other and expressing their responses to disturbances. Recent studies with a laser vibrometer to characterize vibrational response patterns of calm and disturbed bees show that there are significantly different vibration response frequencies of calm and disturbed bees [81]. Our future study will be to integrate a low-cost piezo-electric vibration sensor with the PCB.

Low-cost sensors are subject to gradual drift due to aging and moisture contamination, necessitating periodic recalibration, while biofouling by wax or propolis may alter airflow and measurement accuracy over extended use. While wireless communication was stable in the test location, signal reliability in large or densely distributed apiaries may be affected by distance, interference, and hive placement, motivating alternative communication strategies such as gateway-based or low-power wide-area networks. Moreover, maintenance requirements, including battery replacement intervals, sensor cleaning, and enclosure inspection, represent important operational constraints that should be considered when scaling the system for long-term field applications.

The proposed wireless sensor platform offers a significantly lower cost (approx. $130 per unit vs. $500–$630 for commercial logger HOBO), with comparable accuracy for CO_2_ measurements. The accuracy of the employed sensors for temperature, relative humidity, and CO_2_ concentration was found to be within ~1 °C, ±7–9%, and ±30 ppm, respectively, which is sufficient for apiary monitoring.

### 4.3. Power Supply and Energy Consumption

**Figure 13:**
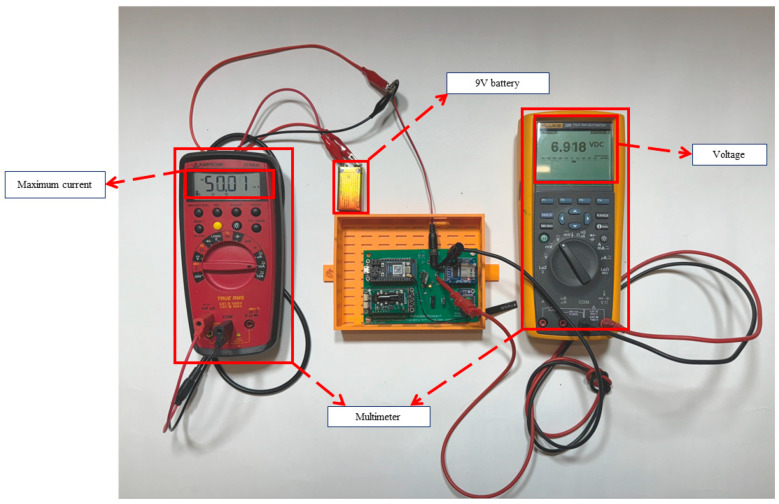
The energy consumption test circuitry.

The proposed PCB’s two designs’ (Ver 3 and Ver 4) energy consumption was tested in the lab conditions using two multimeters and one battery pack, as shown in Figure 13. The voltage drop of the battery of the PCB was tested at different data sampling rates.

**Figure 14:**
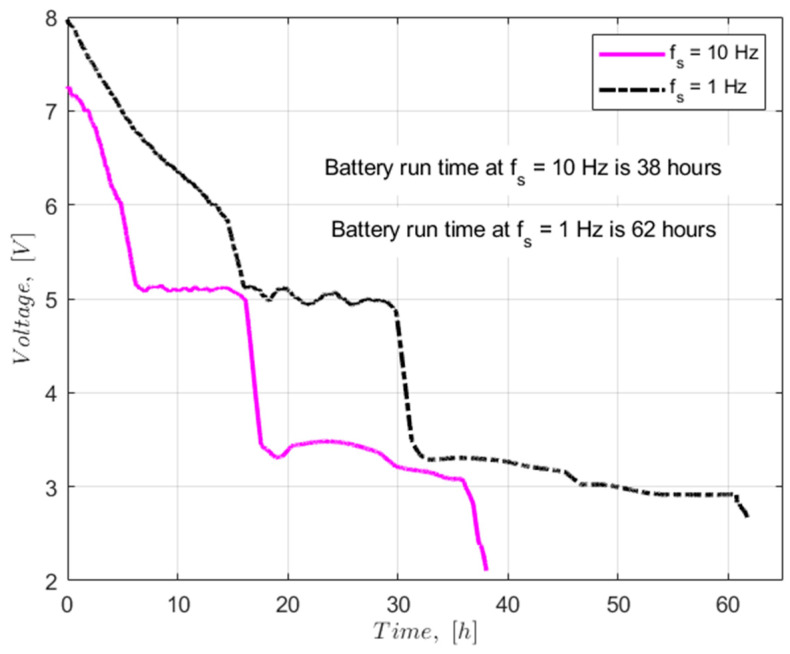
Voltage drops of the power source with two different data sampling frequencies (1 and 10 Hz) with PCB Ver 3.

To optimize energy management, it is essential to consider that the Argon^TM^ Particle microcontroller lacks an efficient low-power mode. Specifically, this microcontroller cannot exit sleep mode deep through a timer; it can only be reactivated from this mode by a high-level signal on pin D8. In voltage consumption test, the proposed PCB system was tested using the battery pack of 9 V DC battery at two different data sampling frequency values, i.e., 1 Hz and 10 Hz—Figure 14. The test results show that, as anticipated, the power consumption at the 10 Hz sampling rate was much higher than the lower data sampling rate of 1 Hz. The battery life with the 10 Hz sampling rate lasted 5479 s, while with 1 Hz, the sampling rate drained the battery of 9 V after 8898 s. In other words, the 10 Hz sampling rate drains the battery power over 60% faster than the 1 Hz data sampling rate.

We measured continuous current draw using a DC Power Analyzer (Agilent N6705B, Agilent Technologies, Santa Clara, CA, USA). For the PCB populated with the SCD30 sensor, we supplied 9 V from Channel 1 and recorded current consumption continuously over 72 h. For the SCD41 sensor, with 6 V from Channel 1 and recorded for 75 h, the SCD41 did not respond reliably at 9 V, necessitating operation at 6 V DC supply. In both experiments, device firmware ran at 0.2 Hz, reflecting the intended low-duty operating mode during deployment.

**Figure 15:**
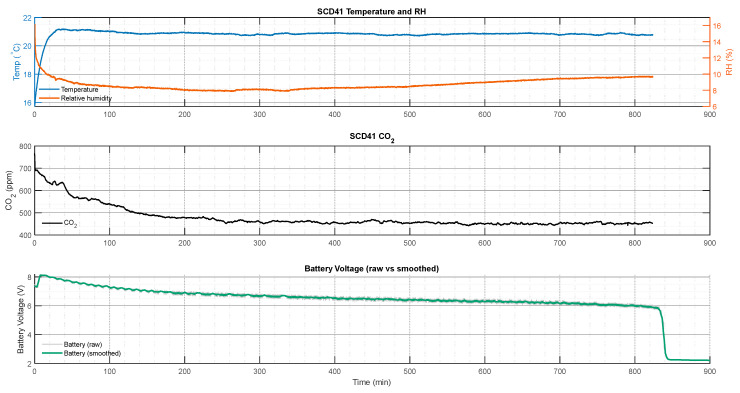
SCD41 sensor readings of temperature, RH, CO_2_ and voltage drops of the battery power source of PCB Ver 4 with 0.01 Hz sampling frequency.

**Figure 16:**
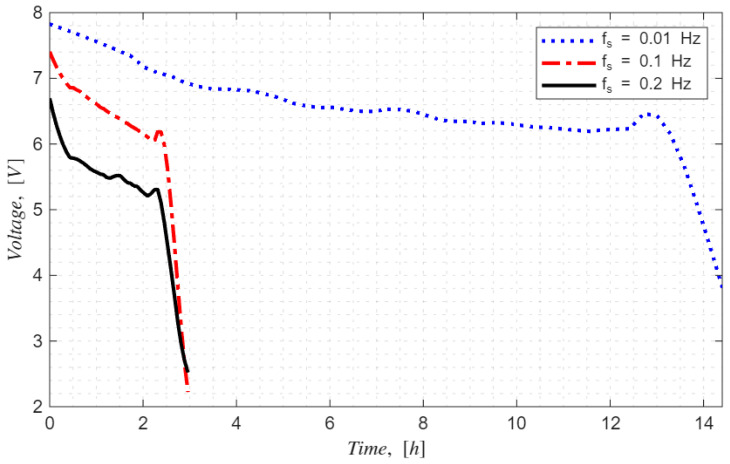
Voltage drop of the battery of the PCB Ver 4 at data sampling frequencies: $f_{s}=\left[0.1,\;0.2,\;0.01\right]\;Hz$.

Figure 15 shows sensor reading consistency and power consumption of SCD41 with 0.01 Hz data sampling rate for approximately 14 h. To evaluate the voltage consumption of benchmarking PCB with SCD41 and SCD30, temperature, humidity, CO_2_, and battery voltage were monitored with different sampling frequencies, e.g., [0.01, 0.1, 0.2] Hz—Figure 16. 

The voltage drop or power consumption of the PCB setup is directly proportional with the data sampling rate, as shown in Figure 14. On the other hand, 0.1 and 0.2 Hz sampling rates resulted in a similar duration of the power source with PCB Ver 4—Figure 16.

Table 4 shows the regression model coefficient values and regression fit model performances, such as $R^{2}$ and *RMSE*, at [0.01, 0.1, 0.2] Hz data sampling frequencies. The found fit models suggest that the exponential decay function can accurately estimate power decay of the battery pack of the designed PCBs and the found fit model quality—$R^{2}$ ranged between 0.85 and 0.90.

The system was designed with modular scalability in mind, allowing multiple units to be deployed simultaneously and managed via a centralized dashboard. We have discussed scalability, including wireless configuration, synchronized data reporting, and user-friendly setup procedures.

### 4.4. Benchmarking Analysis of the Proposed PCB

We have reviewed and analyzed over 70 published works in the domain. Most of the existing research and development works on beehive sensing considered temperature, humidity, and sound with commercially available data loggers and deployed wires for connections, which is inconvenient for installation and for long-term deployment. Our developed system—printed circuit board (PCB)—integrates temperature, humidity, CO_2_ and power management in one single PCB, which is convenient to install and deploy for long-term beehive monitoring purposes. We compiled the most relevant works and compared against our proposed PCB characteristics—Table 5.

### 4.5. Limitations and Challenges

Limited tests: The proposed system needs to be tested for longer periods. In the current study, this has been tested in limited periods during summer (foraging period) and winter (hibernation period), and inside the winter storage, the environmental factors were fully controlled. The container of the beehives had relatively limited airflow.

Housing design: It is not fully validated if the current design of the PCB housing and fasteners is robust enough for longer periods of data collection and in harsh environments, such as transportation of beehives for long distances.

Cloud option: The current version of the system uses ThingSpeak^TM^ (MATLAB R2024b, MathWorks, Natick, MA, USA), and not all necessary data analytics functions have been implemented. There is an alternative option with MQTT needs to be investigated.

## 5. Conclusions

In this study, we demonstrated features and technical capabilities of our proposed PCB to build a WSN system to monitor beehive temperature, CO_2_ gas concentration and relative humidity variables. The initial tests and validation studies show that our designed system is reliable and can be employed for bee colony health monitoring. It should be noted that SCD 41 is energy efficient, but its CO_2_ accuracy range is 400–5000 ppm, with an accuracy of ±40 ppm (+5% of reading). On the other hand, SCD30′s CO_2_ reading range is much larger (400–40,000 ppm), with an accuracy of ±30 ppm (+3% of reading), which is more compatible for beehive monitoring.

In our current study, we have not considered the influence of the measured temperature, relative humidity, and CO_2_ concentration changes on the crop pollination or foraging of bees. These issues will be considered in our next studies when we collect year around observations in different field conditions. During our field tests, the system achieved a data transmission success rate of over 98% under standard Wi-Fi coverage. We have not tried our system in areas with weak signals yet, but during this experiment, data transmission delays of up to 1–2 min were observed when data was syncing in ThingSpeak^TM^ (The MathWorks, Inc., Natick, MA, USA) server, but data buffering ensured that no data was lost. These observations have now been detailed in Section 4.

Future studies will be dedicated to testing the system in a series of beehives for longer periods and during transportation events, including the fall movement of hives from ND to southern CA in preparation for the early spring almond bloom. Moreover, our future research will be aimed at optimizing the housing of the PCB and cloud computing and data analytics options. In addition to optimizing energy efficiency and battery life, we will pursue the options of how to automatically control the sleep mode of the system when the bees are less active. In addition, we will integrate a low-cost piezo-electric vibration sensor to the PCB to measure vibrational responses of bees along with other variables.

## Figures and Tables

**Table 1:** Specifications of the PCB components.

Components	Specifications	Description
Microprocessor	Particle Argon^TM^ (Particle Industries, San Francisco, CA, USA)	
		Wi-Fi and BLE Microcontroller

Co–processor	Espressif ESP32-D0WD(Espressif Systems (Shanghai) Co., Ltd., Shanghai, China)	2.4 GHz Wi-Fi, 802.11 n, up to 150 Mbps
	Nordic Semiconductor nRF52840 SoC (Nordic Semiconductor ASA, Trondheim, Norway)	ARM Cortex-M4F, 64 MHz, 1 MB flash, 256 KB RAM, Bluetooth 5 (2 Mbps, 1 Mbps, 500 Kbps, 125 Kbps)
Additional Features	Li-Po charging RGB LED	Integrated battery connector Status indication
	Reset and Mode buttons	Functional buttons for device operation
	On-board PCB antenna	Integrated for wireless communication
Sensor	Adafruit NDIR SCD30 (Adafruit Industries, New York, NY, USA)	Measures temperature, humidity, and CO_2_
Sensor Specifications	Form factor Measurement range Current consumption	35 mm × 23 mm × 7 mm 400–40,000 ppm 19 mA 1 measurement per 2 s
	Accuracy	±(4–5 °C) (7–9%) (30 ppm + 3%)
Real-Time Clock (RTC)	Adafruit DS3231(Adafruit Industries, New York, NY, USA)	Fast I^2^C interface, low power, 3.3 V operation
Enclosure	Custom 3D-printed box	Designed to accommodate sensors and protect components

**Table 2:** CO_2_ gas concentration readings of LI-850, FRC value, SCD30, and offset values.

Measured Count	1	2	3	4	5
Gas concentration reading in LI-850 Gas Analyzer (µmol mol^−1^)	12,478.2	5369.6	1987.2	767.1	494.8
FRC value in code (ppm)	12,400	5300	1950	700	450
SCD30 output on (ppm)	12,455.7	5634.7	1975.2	752.7	485.9
Offset in SCD30 reading (ppm)	23–24	14–15	12–13	14–15	9–10

**Table 3:** Beehive external disturbance log summary.

Season	Hive	Date	Disturbance Start Time	Disturbance End Time	Notes
Fall 2024	H1	20 September 2024	02:10:05 AM	02:10:58 AM	10 knocks (5 s interval)
	H2	20 September 2024	02:11:18 AM	02:12:15 AM	10 knocks (5 s interval)
	H3	20 September 2024	02:12:35 AM	02:13:35 AM	10 knocks (5 s interval)
Winter 2025	H1	15 January 2025	01:41:08 PM	01:41:58 PM	10 knocks (5 s interval)
	H2	15 January 2025	01:42:18 PM	01:43:13 PM	10 knocks (5 s interval)
	H3	15 January 2025	01:43:33 PM	01:44:23 PM	10 knocks (5 s interval)
	H1	16 January 2025	02:51:18 PM	02:52:13 PM	10 knocks (5 s interval)
	H2	16 January 2025	02:52:21 PM	02:53:27 PM	10 knocks (5 s interval)
	H3	16 January 2025	02:53:37 PM	02:54:29 PM	10 knocks (5 s interval)
Fall 2025	H1	24 September 2025	03:13:15 AM	03:14:03 AM	10 knocks (5 s interval)
	H2	24 September 2025	03:15:16 AM	03:16:01 AM	10 knocks (5 s interval)
	H1	25 September 2025	12:38:11 AM	12:39:03 AM	10 knocks (5 s interval)
	H2	25 September 2025	12:41:13 AM	12:42:27 AM	10 knocks (5 s interval)
	H1	26 September 2025	02:45:38 AM	02:46:58 AM	10 knocks (5 s interval)
	H2	26 September 2025	02:50:48 AM	02:51:58 AM	10 knocks (5 s interval)

**Table 4:** Regression model values of voltage drop at 0.2, 0.1, and 0.01 Hz data sampling rates.

$\mathrm{Data}\;\mathrm{Sampling}\;\mathrm{Frequency},\;f_{s}$	$\mathrm{Model}:\;V\left(t\right)=V_{0}-V_{1}t-e^{-\left(V_{d}\left(t-t_{d}\right)\right)}$	$R^{2}$	RMSE
0.2 Hz	$V\left(t\right)=6.55-0.77t-e^{-\left(5.26\left(t-2.96\right)\right)}$	0.89	0.31
0.1 Hz	$V\left(t\right)=7.50-0.84t-e^{-\left(9.02\left(t-2.96\right)\right)}$	0.90	0.40
0.01 Hz	$V\left(t\right)=7.55-0.13t-e^{-\left(1.95\left(t-14.45\right)\right)}$	0.85	0.28

**Table 5:** Comparison of similar development works for beehive monitoring with the proposed PCB design.

Reference	Key Contributions	Limitations	Advantages of the Proposed System
[8,16,17,25,26,29,35,39,41,74,77]	Wireless beehive monitoring with temperature, humidity, nectar flow analysis, weight-based inference of behavior and cloud data storage.	Focuses on hive weight monitoring; limited environmental sensing inside hive.	Proposed PCB integrates CO_2_ sensing with environmental variables for accurate hive monitoring.
[9,21]	WSN system to monitor bee incoming/outgoing activities and environmental factors in real time with ≈93.9% counting accuracy.	Focuses on bee activity counting and basic environmental sensing; does not monitor gas concentration inside the hive.	Proposed PCB integrates CO_2_ monitoring with environmental sensing, providing deeper insights into accuracy and power consumption.
[10]	IoT-enabled beehive monitoring system with decision-tree algorithm to classify hive conditions (95.38% accuracy) and predict weather patterns.	Complex multi-sensor system with relatively high energy consumption that requires frequent battery change.	PCB is compact and low-power hungry sensor architecture, which is suitable for long-term deployment.
[15,34,42,67]	Raspberry-Pi-based WSN for continuous monitoring of hive temperature, humidity, and acoustic activity with real-time data visualization.	It does not include gas sensing, and its cost is high.	PCB includes CO_2_ monitoring.
[23]	A solar-powered smart beehive network for long-term monitoring and predictive analysis of honey robbing behavior using multi-hive datasets.	Behavioral analysis and predictive modeling; it does not emphasize detailed internal hive gas monitoring.	Proposed system integrates CO_2_ sensing with environmental monitoring, enabling deeper understanding of hive ventilation, respiration, and colony health.
[49]	Investigated system to monitor how honey bee colonies regulate internal CO_2_ concentration and temperature despite changes in hive ventilation; continuous CO_2_ monitoring at 1 s intervals revealed strong daily cycles and concentrations exceeding 11,000 ppm.	System designed mainly for experimental research, lacking IoT connectivity or real-time remote monitoring capability.	PCB integrates T, RH and CO_2_ sensing with IoT-based wireless monitoring, enabling real-time remote assessment of hive environmental conditions and colony health.

## Data Availability

The original contributions presented in this study are included in the article. Further inquiries can be directed to the corresponding author.

## Abbreviations

The following abbreviations are used in this manuscript:

WSN	Wireless Sensor Network
PCB	Printed Circuit Board
NDIR	Non-Dispersive Infrared
RFID	Radio Frequency Identification
IDE	Integrated Development Environment
USDA	United States Department of Agriculture
ARS	Agricultural Research Service
